# Association of Lower Extremity Lymphedema With Physical Functioning and Activities of Daily Living Among Older Survivors of Colorectal, Endometrial, and Ovarian Cancer

**DOI:** 10.1001/jamanetworkopen.2022.1671

**Published:** 2022-03-09

**Authors:** Xiaochen Zhang, Eric M. McLaughlin, Jessica L. Krok-Schoen, Michelle Naughton, Brittany M. Bernardo, Andrea Cheville, Matthew Allison, Marcia Stefanick, Jennifer W. Bea, Electra D. Paskett

**Affiliations:** 1 Division of Cancer Prevention and Control, Department of Internal Medicine, The Ohio State University, Columbus; 2 Center for Biostatistics, The Ohio State University, Columbus; 3 Division of Health Sciences, School of Health and Rehabilitation Sciences, The Ohio State University, Columbus; 4 Department of Physical Medicine and Rehabilitation, Mayo Clinic, Rochester, Minnesota; 5 Department of Family Medicine and Public Health, University of California San Diego, La Jolla; 6 Department of Obstetrics and Gynecology, Stanford University, Stanford, California; 7 Department of Health Promotion Sciences, University of Arizona, Tucson

## Abstract

**Importance:**

Lower extremity lymphedema (LEL) is associated with decreased physical functioning (PF) and activities of daily living (ADLs) limitations. However, the prevalence of LEL in older survivors of cancer is unknown.

**Objectives:**

To examine LEL among older female survivors of colorectal, endometrial, or ovarian cancer and investigate the association of LEL with PF and ADLs.

**Design, Setting, and Participants:**

This secondary analysis of the Women's Health Initiative (WHI) Life and Longevity After Cancer (LILAC) study was conducted using data from postmenopausal women enrolled at 40 US centers. Participants were women who had a prior diagnosis of endometrial, colorectal, or ovarian cancer and who had completed the WHI LILAC baseline and year 1 follow-up questionnaires as of September 2017.

**Exposures:**

The 13-item Lower Extremity Lymphedema Screening Questionnaire in Women was used to determine LEL (ie, score ≥5).

**Main Outcomes and Measures:**

Validated surveys were used to assess PF and ADLs.

**Results:**

Among 900 older women diagnosed with endometrial, colorectal, or ovarian cancer, the mean (SD) age was 78.5 (5.9) years and the mean (range) time since cancer diagnosis was 8.75 (1.42-20.23) years. Overall, 292 women (32.4%) reported LEL, with the highest LEL prevalence among survivors of ovarian cancer (38 of 104 women [36.5%]), followed by survivors of endometrial cancer (122 of 375 women [32.5%]) and colorectal cancer (132 of 421 women [31.4%]). Compared with women without LEL, women with LEL had a PF score that was lower by a mean (SE) 16.8 (1.7) points (*P* < .001) and higher odds of needing help with ADLs (odds ratio [OR], 2.45; 95% CI, 1.64-3.67). In the association of LEL with PF, the mean (SE) decrease in PF score was greatest among survivors of colorectal cancer (−21.8 [2.6]) compared with survivors of endometrial cancer (−13.3 [2.7]) and ovarian cancer (−12.8 [5.2]). Additionally, among survivors of colorectal cancer, LEL was associated with increased odds of needing help with ADLs (OR, 3.59; 95% CI, 1.94-6.66), while there was no such association among survivors of endometrial cancer or ovarian cancer. However, there were no interaction associations between LEL and cancer type for either outcome. Additionally, the overall mean (SE) difference in PF between women with and without LEL was greater among those aged 80 years and older (−19.4 [2.6] points) vs those aged 65 to 79 years (−14.9 [2.2] points). However, among survivors of colorectal cancer, the mean (SE) difference in PF score was greater among women aged 65 to 79 years (−22.9 [3.7] points) vs those aged 80 years or older (−20.8 [3.7] points) (*P* for 3-way interaction = .03).

**Conclusions and Relevance:**

This study found that nearly one-third of older female survivors of colorectal, endometrial, or ovarian cancer experienced LEL and that LEL was associated with decreased PF and increased odds of needing help with ADLs. These findings suggest that clinicians may need to regularly assess LEL among older survivors of cancer and provide effective interventions to reduce LEL symptoms and improve PF for this population.

## Key Points

**Question:**

Is lower extremity lymphedema (LEL) associated with decreased physical functioning and activities of daily living among older survivors of colorectal, endometrial, and ovarian cancer?

**Findings:**

In this cohort study of 900 older female survivors of endometrial, colorectal, or ovarian cancer, nearly one-third of survivors reported LEL. It was associated with decreased physical functioning and higher odds of needing help with activities of daily living, with the largest changes among women aged 80 years or older or women with colorectal cancer.

**Meaning:**

These findings suggest that clinicians may need to regularly assess and manage LEL among survivors of cancer to improve physical functioning.

## Teaser

This cohort study investigates the association of lower extremity lymphedema with physical functioning and the need for assistance with activities of daily living among older female survivors of colorectal, endometrial, and ovarian cancer.

## Introduction

Each year, approximately 158 000 women are diagnosed with endometrial, ovarian, or colorectal cancer.^1^ Given that survivors of cancer live longer because of early detection and effective treatments, long-term sequelae after cancer treatment and quality of life have become critical to survivors of cancer. One common sequela for survivors of gynecologic and colorectal cancer after surgeries involving lymph node dissection or pelvic radiotherapy is lower extremity lymphedema (LEL).^2,3,4,5,6,7,8,9,10,11^ This outcome is characterized by abnormal accumulation of protein-rich interstitial fluid, often due to obstruction or scarring of the lymphatic system.^12,13,14^ LEL can present as edema in the legs, feet, lower abdomen, hips, and genitals and can develop within months to several years after an individual completes cancer treatment.^6,11,15,16,17^

The reported prevalence of LEL fluctuates dramatically because of variation in diagnostic criteria, timing of assessment, measurement techniques, potential reporting bias, and follow-up time.^18,19,20^ It is commonly estimated that 20% to 60% of survivors of gynecologic cancer have LEL,^5,21,22,23,24^ whereas the reported prevalence ranges from 2.4% to 78%.^2,4,19,25,26,27,28^ The prevalence of LEL varies by cancer site, ranging from 1% to 47% in endometrial cancer,^5,29,30^ 0% to 81% in cervical cancer,^5,31,32^ and 5% to 41% in ovarian cancer.^4,5,33,34^ Notably, the prevalence of LEL in colorectal cancer is unknown because of the lack of published studies.

LEL is a chronic, incurable condition with symptoms including swelling, heaviness, pain and discomfort, lower physical functioning (PF), decreased mobility to perform activities of daily living (ADLs), and increased psychological concerns.^4,25,26,27,35,36,37^ Survivors of cancer who have LEL experience a decline in the ability to walk, lift heavy objects, or stand for long periods, and these declines are associated with increased supportive care needs and decreased quality of life.^2,4,26,27,36,38,39^ However, the association of LEL with decreased physical activity and mobility to perform ADLs among older survivors of cancer is understudied. Because of age-associated PF decline, it is possible that older survivors of cancer with LEL may experience additional challenges in PF and interference with ADLs compared with those without LEL.^40^

This study aimed to examine LEL among older female survivors of long-term (ie, >5 years since cancer diagnosis) endometrial, colorectal, or ovarian cancer using a validated questionnaire for detecting clinically relevant LEL among women.^41^ The study also aimed to investigate the association of LEL with PF and interference with ADLs.

## Methods

Data from the Women's Health Initiative (WHI) and the WHI Life and Longevity after Cancer (LILAC) Study, an ancillary study to the WHI, were used in this cohort study.^42^ Written informed consent was obtained from all participants. The WHI study was approved by the Fred Hutchinson Cancer Research Center's institutional review board, and the WHI LILAC study was approved at the 3 institutions of the multiple principal investigators (ie, the Fred Hutchinson Cancer Research Center, Kaiser-Permanente Northern California, and The Ohio State University). This cohort study followed the Strengthening the Reporting of Observational Studies in Epidemiology (STROBE) reporting guideline.

Study details have been described previously.^42,43^ Briefly, 161 808 postmenopausal women (aged 50-79 years) were enrolled between 1993 and 1998 at 40 clinical centers into 1 or more randomized clinical trials (68 132 women in WHI-CTs) or an observational study (93 676 women in WHI-OS). Participants were followed up for up to 10 years in WHI, and many participants continued follow-up in WHI to the present. In 2013, the WHI LILAC study began enrolling WHI participants who had been previously diagnosed with selected cancers (ie, breast, endometrial, ovarian, lung, and colorectal cancers, melanoma, lymphoma, and leukemia).^42^ LILAC participants completed baseline, year 1, and year 2 follow-up questionnaires.^44^ The sample for this cohort study included LILAC participants who had a diagnosis of endometrial, colorectal, or ovarian cancer and who had completed WHI LILAC baseline and year 1 follow-up questionnaires as of September 2017.

Lower extremity lymphedema was self-reported on the LILAC year 1 questionnaire using the 13-item Lower Extremity Lymphedema Screening Questionnaire in Women.^41^ Women were asked about symptoms in the lower part of the body in the previous 4 weeks, including tightness, heaviness, pain or discomfort, and swelling in the leg, ankle, foot, hip, below-stomach, and genital areas. Response categories were “not at all” (0), “a little bit” (1), “somewhat” (2), “quite a bit” (3), and “very much” (4). Total LEL scores range from 0 to 52, with scores of 5 or more indicating LEL with high sensitivity (95%) and specificity (86%).^41^ Women were also asked whether swelling interfered with their daily activities (yes or no), and if so, the type of activities (ie, employment, recreation, rest or sleep, housework or gardening, social activities, and other).

A 10-item PF assessment was taken from the WHI annual follow-up questionnaire (ie, the PF subscale of the RAND 36-Item Health Survey).^45^ Participants were asked, “Does your health now limit you in these activities, and if so, how much?” Activities included vigorous activities (eg, running, lifting heavy objects, and strenuous sports), moderate activities (eg, moving a table, vacuuming, bowling, and golfing), carrying groceries, climbing 1 or several flights of stairs, walking more than a mile, walking 1 or several blocks, bathing or dressing oneself, and bending, kneeling, or stooping. Response options were “not limited at all” (3), “limited a little” (2), and “limited a lot” (1). PF subscale scores ranged from 0 (low functioning) to 100 (high functioning).

A 6-item ADL scale was also assessed on the WHI annual follow-up questionnaire, derived from the Lawton Instrumental Activities of Daily Living and Katz Index of Independence in Activities of Daily Living.^46,47^ Participants were asked how much help if any they needed to do routine activities, including feeding themselves, dressing themselves, getting in and out of bed, taking a bath or shower, doing their own grocery shopping, and keeping track of and taking medicines. Participants could choose from “by myself without help” (1), “with some help” (0.5), and “completely unable to do by myself” (0). Composite ADL score ranges from 0 to 6. A score of 6 indicates that the participant was able to do all activities alone with no help, whereas scores of 5 or less indicate needing some help or being unable to perform at least 1 ADL.

Data on age at LILAC year 1 questionnaire, education, race, ethnicity, marital status, insurance type, body mass index (BMI; calculated as weight in kilograms divided by height in meters squared), and receipt of chemotherapy or radiation were self-reported. Race and ethnicity were asked separately. Participants were asked whether they were Spanish, Hispanic, or Latino (hereafter, *Hispanic or Latino*). Participants could choose more than 1 racial group from among American Indian or Alaska Native, Asian (ie, Asian Indian, Chinese, Filipino, Japanese, Korean, Vietnamese, or other Asian), Black or African American, Native Hawaiian or other Pacific Islander (ie, Guamanian or Chamorro, Samoan, or other Pacific Islander), White, or other or not specified race. Participants who reported more than 1 racial group were combined as "more than 1 race." Race and ethnicity were assessed within a sociopolitical framework as proxies for historical and ongoing differences in social determinants of health, which are associated with the severity of diseases and symptoms and quality of life. Cancer stage at diagnosis and number of lymph nodes examined or removed and time at diagnosis were collected from medical records.

### Statistical Analysis

The percentage of participants who reported LEL (ie, LEL score ≥5) was assessed overall and by cancer site. The mean PF score was compared between participants with LEL scores less than 5 (ie, no LEL) and those who reported LEL scores of 5 or more. For participants who reported LEL, the frequency of swelling that interfered with ADLs and the type of activities involved were summarized. The proportion of individuals needing help with ADLs (ie, ADL score ≤5) was compared between participants who reported no LEL vs those who reported LEL.

Linear regression was used to investigate the association between LEL and PF score overall and stratified by cancer type. Least square (LS) means with standard errors (SEs) and 95% CIs were estimated for unadjusted and adjusted models (controlled for age, education, race and ethnicity, marital status, insurance type at enrollment, BMI, cancer stage at diagnosis, number of lymph nodes examined, time since diagnosis, self-reported chemotherapy, and self-reported receipt of radiation). A sensitivity analysis with median regression was used to account for the skewed distribution of the PF score. Because needing help with ADLs was defined as an ADL score of 5 or less, logistic regression models were used to calculate unadjusted and adjusted odds ratios (ORs) and 95% CIs for the association between LEL and needing help with ADLs for all cancer sites combined and stratified by cancer type.

All statistical tests were 2-sided. The level of significance was *P* ≤ .05. Additional analyses examined the association between LEL and PF and needing help with ADLs by cancer type. Given that BMI and age may modify the association of LEL with PF and needing help with ADLs, further stratification by BMI category (ie, <25, 25-29.9, and ≥30) and age group (ie, ages 65-79 and ≥80 years) were conducted separately. Adjusted Wald tests were used to examine these interactions. All analyses were performed from April 28 to October 14, 2020, using SAS statistical software version 9.4 (SAS Institute).

## Results

There were 1667 women enrolled in LILAC with endometrial, colorectal, or ovarian cancer. A total of 767 women were excluded for the following reasons: 3 women with cancer prior to WHI enrollment, 110 women with other cancers diagnosed during follow-up, 282 women with missing questionnaires, 57 women with no data on PF or ADLs, and 315 women with unknown lymphedema score. The final analysis cohort included 900 women. Compared with those who were included, participants who were excluded from the current analysis were older; more likely to be widowed, have high school or less education, and have colorectal cancer; and less likely to have private health insurance (eTable 1 in the Supplement).

Among 900 women included in our analysis, 421 women (46.8%) were diagnosed with colorectal cancer, 375 women (41.7%) with endometrial cancer, and 104 women (11.6%) with ovarian cancer. The mean (SD; range) age at LILAC year 1 survey was 78.5 (5.9; 65.0-96.0) years (Table 1). The mean (range) time since cancer diagnosis was 8.75 (1.42-20.23) years. The racial breakdown of participants was as follows: 1 American Indian or Alaska Native woman (0.1%), 19 Asian women (2.1%), 33 Black or African American women (3.7%), 827 White women (91.9%), 11 women with more than 1 race (1.2%), and 9 women with other or not specified race (1%). There were 878 women who self-identified as not Hispanic or Latino (97.6%), 20 women who self-identified as Hispanic or Latino (2.2%), and 2 women with unknown or not reported ethnicity (0.2%). Most women were married and had some college education and private insurance. The mean (SD) BMI at LILAC year 1 survey was 26.8 (6.0). In terms of cancer characteristics, there were 585 women (65.2%) with an in situ or localized stage of cancer, 350 women (38.9%) with a cancer diagnosis more than 10 years in the past, and 644 women (71.6%) with 5 or more lymph nodes removed.

**Table 1.** Demographic and Clinical Characteristics by LEL Status

Characteristic	Women, No. (%)			*P* value
	Overall (N = 900)	LEL		
		No (n = 608)^a^	Yes (n = 292)^b^	
Age, mean (SD), y	78.5 (5.9)	78.1 (5.7)	79.3 (6.1)	.002
Race^c^				
American Indian or Alaska Native	1 (0.1)	1 (0.2)	0	.99
Asian	19 (2.1)	11 (1.8)	8 (2.7)	
Black or African American	33 (3.7)	22 (3.6)	11 (3.8)	
Native Hawaiian or other Pacific Islander	0	0	0	
White	827 (91.9)	559 (91.9)	268 (91.8)	
≥1 race	11 (1.2)	11 (1.8)	0	
Other not specified	9 (1.0)	4 (0.7)	5 (1.7)	
Ethnicity^d^				
Not Hispanic or Latino	878 (97.6)	595 (97.9)	283 (96.9)	.47
Hispanic or Latino	20 (2.2)	12 (2.0)	8 (2.7)	
Marital status				
Married or living as married	409 (47.6)	293 (50.3)	116 (41.7)	.01
Widowed	303 (35.2)	192 (33.0)	111 (39.9)	
Divorced or separated	109 (12.7)	77 (13.2)	32 (11.5)	
Never married	39 (4.5)	20 (3.4)	19 (6.8)	
Education				
College or associate’s degree	766 (85.4)	523 (86.4)	243 (83.2)	.20
≤High school	131 (14.6)	82 (13.6)	49 (16.8)	
Insurance				
Private	626 (70.1)	432 (71.5)	194 (67.1)	.02
Public	50 (5.6)	24 (4.0)	26 (9.0)	
Public and private	181 (20.3)	123 (20.4)	58 (20.1)	
No insurance	36 (4.0)	25 (4.1)	11 (3.8)	
BMI, mean (SD)	26.8 (6.0)	26.2 (5.7)	28.2 (6.4)	<.001
Cancer type^e^				
Colorectal	421 (46.8)	289 (68.6)	132 (31.4)	.60
Endometrial	375 (41.7)	253 (67.5)	122 (32.5)	
Ovarian	104 (11.6)	66 (63.5)	38 (36.5)	
Stage				
In situ or localized	585 (65.2)	403 (66.4)	182 (62.8)	.16
Regional	252 (28.1)	170 (28.0)	82 (28.3)	
Distant	60 (6.7)	34 (5.6)	26 (9.0)	
Time since diagnosis, y				
<5	277 (30.8)	186 (30.6)	91 (31.2)	.47
5-10	272 (30.3)	177 (29.2)	95 (32.5)	
>10	350 (38.9)	244 (40.2)	106 (36.3)	
Lymph nodes examined, No.				
0	197 (21.9)	134 (22.1)	63 (21.6)	.82
1-4	58 (6.5)	37 (6.1)	21 (7.2)	
≥5	644 (71.6)	436 (71.8)	208 (71.2)	
Treatment				
Radiation therapy	141 (15.8)	85 (14.1)	56 (19.4)	.04
Chemotherapy	298 (33.3)	187 (30.8)	111 (38.4)	.02
Hormone therapy	17 (1.9)	10 (1.7)	7 (2.5)	.43
Cancer recurrence				
No	829 (92.1)	563 (92.6)	266 (91.1)	.44
Yes	71 (7.9)	45 (7.4)	26 (8.9)	

Abbreviations: BMI, body mass index (calculated as weight in kilograms divided by height in meters squared); LEL, lower extremity lymphedema.

^a^ Defined as a score of 1 to 4 in the 13-item Lower Extremity Lymphedema Screening Questionnaire in Women.

^b^ Defined as a score of 5 or more in the 13-item Lower Extremity Lymphedema Screening Questionnaire in Women.

^c^ Participants could choose more than 1 racial group from among American Indian or Alaska Native, Asian (ie, Asian Indian, Chinese, Filipino, Japanese, Korean, Vietnamese, or other Asian), Black or African American, Native Hawaiian or other Pacific Islander (ie, Guamanian or Chamorro, Samoan, or other Pacific Islander), White, or other or not specified race. Participants who reported more than 1 racial group were combined as "more than 1 race."

^d^ Participants were asked whether they were Spanish, Hispanic, or Latino.

^e^ Percentages are for row totals.

At the LILAC year 1 survey, the mean (SD) self-reported LEL score was 4.7 (7.0). Overall, 292 participants (32.4%) reported LEL (Table 2), with the highest proportion among women with ovarian cancer (38 women [36.5%]), followed by those with endometrial cancer (122 women [32.5%]) and those with colorectal cancer (132 women [31.4%]) (Table 1). Women who were older, widowed, had public insurance, had higher BMI, and had radiation or chemotherapy were more likely to report LEL. Among women who self-reported LEL, 63 women (22.6%) reported that swelling interfered with daily activities, such as housework (40 women [14.3%]), rest or sleep (34 women [12.2%]), and recreation (26 women [9.3%]).

**Table 2.** PF, Swelling Interference With Daily Activity, and ADLs by LEL Status

Outcome	Women, No. (%)			*P* value
	Overall (N = 900)	LEL		
		No^a^	Yes^b^	
LEL score, mean (SD)	4.7 (7.0)	1.0 (1.3)	12.3 (7.8)	<.001
PF score^c^				
Mean (SD)	66.3 (27.2)	72.8 (24.1)	52.8 (28.6)	<.001
Category				
<40	165 (18.3)	72 (11.8)	93 (31.9)	<.001
40-69	209 (23.2)	117 (19.3)	92 (31.5)	
≥70	526 (58.4)	419 (68.9)	107 (36.6)	
Swelling interference with daily activity^d^			63 (22.6)	
Employment	NA	NA	2 (0.7)	NA
Recreation	NA	NA	26 (9.3)	
Rest or sleep	NA	NA	34 (12.2)	
Housework	NA	NA	40 (14.3)	
Social activities	NA	NA	19 (6.8)	
Other	NA	NA	8 (2.9)	
ADLs				
0-5 (need at least some help)	140 (15.6)	65 (10.7)	75 (25.7)	<.001
6 (all activities with no help)	760 (84.4)	543 (89.3)	217 (74.3)	

Abbreviations: ADLs, activities of daily living; LEL, lower extremity lymphedema; NA, not applicable; PF, physical function.

^a^ Defined as a score of 1 to 4 in the 13-item Lower Extremity Lymphedema Screening Questionnaire in Women.

^b^ Defined as a score of 5 or more in the 13-item Lower Extremity Lymphedema Screening Questionnaire in Women.

^c^ PF score calculated using RAND-36 physical function subscale.

^d^ Totals for interference may not add up to 100% because women may have had swelling interfere with more than 1 specific daily activity. No *P* value is given for LEL score for interference given that the categorical variable is based on LEL score.

Overall, the mean (SD) PF score was 66.3 (27.2) (Table 2), with a significantly higher score reported among women without LEL compared with those who reported LEL (72.8 [24.1] vs 52.8 [28.6]; *P* < .001). Compared with those without LEL, women with LEL had unadjusted and adjusted PF scores that were a mean (SE) 20.0 (1.8) points lower (*P* < .001) and 16.8 (1.7) points lower (*P* < .001), respectively (Table 3). After adjusting for covariates, comparing women who reported LEL with women who did not, mean (SE) differences in PF score were greater among women who had a diagnosis of colorectal cancer (−21.8 [2.6] points), followed by women with endometrial cancer (−13.3 [2.7] points), and women with ovarian cancer (−12.8 [5.2] points). However, there was no interaction association between LEL and cancer type.

**Table 3.** Association Between LEL and PF

PF score^b^	Women with LEL, No. (%)		Unadjusted		Adjusted^a^		Age 65-79 y		Age ≥80 y		*P* value for interaction^c^
	Yes	No	LS mean (SE) [95% CI], points	*P* value	LS mean (SE) [95% CI], points	*P* value	aLS mean (SE) [95% CI], points^a^	*P* value	aLS mean (SE) [95% CI], points^a^	*P* value	
Overall	292 (32.4)	608 (67.6)	−20.0 (1.8) [−23.8 to −16.4]	<.001	−16.8 (1.7) [−20.1 to −13.4]	<.001	−14.9 (2.2) [−19.3 to −10.6]	<.001	−19.4 (2.6) [−24.5 to −14.2]	<.001	.19
By cancer site											
Colorectal	132 (31.4)	289 (68.6)	−25.3 (2.6) [−30.5 to −20.2]	<.001	−21.8 (2.6) [−27.0 to −16.7]	<.001	−22.9 (3.7) [−30.1 to −15.7]	<.001	−20.8 (3.7) [−28.0 to −13.5]	<.001	.68
Endometrial	122 (32.5)	253 (67.5)	−15.0 (2.9) [−20.7 to −9.2]	<.001	−13.3 (2.7) [−18.6 to −8.0]	<.001	−9.9 (3.3) [−16.4 to −3.4]	.003	−19.8 (4.7) [−28.9 to −10.6]	<.001	.09
Ovarian	38 (36.5)	66 (63.5)	−18.3 (4.9) [−28.0 to −8.7]	<.001	−12.8 (5.2) [−23.1 to −2.5]	.02	−9.1 (6.7) [−22.5 to 4.3]	.18	−18.1 (8.0) [−34.0 to −2.2]	.03	.39

Abbreviations: aLS, adjusted least square; LEL, lower extremity lymphedema; LS, least square; PF score, Physical Function score.

^a^ Adjusted model contains covariates, including age, body mass index (calculated as weight in kilograms divided by height in meters squared), education, race and ethnicity, marital status, insurance type at enrollment, cancer stage at diagnosis, number of lymph nodes examined, time since diagnosis, self-reported chemotherapy, and self-reported radiation. Race and ethnicity were grouped here as White, Black or African American, and other race or ethnicity because of small sample sizes.

^b^ Linear regression was used to estimate the mean difference of PF score between women who reported LEL and those who did not report LEL. The interaction association of LEL and cancer type had a *P* value of .16.^c^Adjusted Wald test was used to test the interaction association. *P* value for the 3-way interaction of LEL by age group by cancer type was .03.

When stratified by age group, mean (SE) PF score difference between women reporting LEL and those not reporting LEL was greater among women aged 80 years and older (−19.4 [2.6] points) than among women aged 65 to 79 years (−14.9 [2.2] points) (Table 3). A similar pattern was observed among women who had a diagnosis of endometrial or ovarian cancer. Among women with endometrial cancer, the mean (SE) PF score difference by LEL was −9.9 (3.3) points among women aged 65 to 79 years and −19.8 (4.7) points among women aged 80 years or older. Among women with ovarian cancer, the mean (SE) PF score difference by LEL was −9.1 (6.7) points among women aged 65 to 79 years and −18.1 (8.0) points among women aged 80 years or older. However, among women who had a diagnosis of colorectal cancer, the mean (SE) PF score difference by LEL was greater among women aged 65 to 79 years (−22.9 [3.7] points) than among women aged 80 years old or older (−20.8 [3.7] points). The 3-way interaction of age by LEL by cancer type was significant (*P* = .03). However, when stratified by BMI category, the magnitude of the difference in mean PF score in the association between LEL and PF score did not differ, and the 3-way interaction of BMI category by LEL by cancer type was not significant (eTable 2 in the Supplement).

Overall, 140 women (15.6%) needed at least some help with ADLs (Table 2). Women who reported LEL were more likely to need help with ADLs compared with 608 women who reported no LEL (75 women [25.7%] vs 65 women [10.7%]; *P* < .001). Women who reported LEL had higher odds of needing help with ADLs (adjusted OR, 2.45; 95% CI, 1.64-3.67) (Table 4) compared with those who did not report LEL. After adjusting for covariates, among women with a diagnosis of colorectal cancer, those who reported LEL had higher odds of needing help with ADLs (OR. 3.59, 95% CI: 1.94-6.66) compared with those who did not report LEL. However, these associations were not significant among women with a diagnosis of endometrial or ovarian cancer. There was no interaction association of LEL and cancer type.

**Table 4.** Association Between LEL and ADLs

Need help with ADLs^b^	Women with LEL, No. (%)		Unadjusted		Adjusted^a^		Age 65-79 y		Age ≥80 y		*P* interaction^c^
	Yes	No	OR (95% CI)	*P* value	OR (95% CI)	*P* value	aOR (95% CI)^a^	*P* value	aOR (95% CI)^a^	*P* value	
Overall	292 (32.4)	608 (67.6)	2.89 (2.00-4.17)	<.001	2.45 (1.64-3.67)	<.001	2.02 (1.13-3.61)	.02	3.39 (1.99-5.79)	<.001	.20
By cancer site											
Colorectal	132 (31.4)	289 (68.6)	4.17 (2.47-7.05)	<.001	3.59 (1.94-6.66)	<.001	2.44 (0.94-6.32)	.07	5.64 (2.62-12.16)	<.001	.17
Endometrial	122 (32.5)	253 (67.5)	1.94 (1.09-3.44)	.02	1.88 (0.96-3.70)	.07	1.64 (0.70-3.86)	.26	2.55 (0.95-6.82)	.06	.51
Ovarian	38 (36.5)	66 (63.5)	2.91 (0.77-11.05)	.12	3.53 (0.59-21.20)	.19	4.17 (0.22-78.00)	.34	2.22 (0.20-24.75)	.52	.74

Abbreviations: ADLs, activities of daily living; aOR, adjusted odds ratio; LEL, lower extremity lymphedema; OR, odds ratio.

^a^ Adjusted model contains covariates, including age, body mass index (calculated as weight in kilograms divided by height in meters squared), education, race and ethnicity, marital status, insurance type at enrollment, cancer stage at diagnosis, number of lymph nodes examined, time since diagnosis, self-reported chemotherapy, and self-reported radiation. Race and ethnicity were grouped here as White, Black or African American, and other race or ethnicity because of small sample sizes.

^b^ Logistic regression was used to estimate odds of needing help with ADLs (ie, score ≤5) among women who reported LEL vs those who did not report LEL. The interaction association of LEL and cancer type had a *P* value of .19.

^c^ Adjusted Wald test was used to test the interaction association. The *P* value for the 3-way interaction association of LEL by age group by cancer type was .22.

When further stratified by age group, comparing women who reported LEL with those who did not, the OR for needing help with ADLs was greater among women aged 80 years or older (OR, 3.39; 95% CI, 1.99-5.79) than among women aged 65 to 79 years (OR, 2.03; 95% CI, 1.13-3.61) (Table 4). A similar pattern was observed among women who had a diagnosis of colorectal cancer. Among women who had a diagnosis of endometrial or ovarian cancer, no statistically significant difference in odds of needing help with ADLs was observed by age group. There was no 3-way interaction association of age by LEL by cancer type. When stratified by BMI category, there was no interaction association of LEL by BMI category, and there was no 3-way interaction association of LEL by BMI category by cancer type (eTable 2 in the Supplement).

## Discussion

This cohort study examined LEL among older female survivors of cancer with colorectal, endometrial, and ovarian cancer and investigated the association of LEL with PF and ADLs. We found that nearly one-third of older survivors reported LEL. Compared with women who did not report LEL, those who reported LEL experienced substantially lower PF and were more likely to need help with ADLs. These findings are consistent with those of previous studies.^26,35,36,37^ Additionally, in the association of LEL with ADLs, ORs were greater among women aged 80 years or older compared with among women aged 65 to 79 years. Our findings suggest the urgent need to identify and implement interventions, especially among older survivors of cancer, to reduce LEL symptoms, improve PF, and maintain or improve ability to perform ADLs. Given that performing ADLs is critical to maintaining independent living and quality of life, addressing limitations in performing ADLs, especially for older survivors of cancer, should be a core of clinical management.^48,49,50^

Moreover, we examined self-reported LEL among women who had a diagnosis of colorectal cancer, which expanded LEL research from gynecologic cancer in previous reports.^2,5,15,19,21,25,27,29,31,34,36,38^ Although there was no interaction of cancer type on the association of LEL and PF, we observed a greater difference in PF between those who reported LEL compared with those who did not in colorectal cancer than in endometrial and ovarian cancer. Similarly, the OR of needing help with ADLs among those who reported LEL compared with those who did not report LEL was greater in colorectal cancer than other cancers. These findings suggest that clinicians may want to consider conducting regular clinical assessments to identify LEL among survivors of cancer, especially survivors of colorectal cancer, to provide resources (eg, physical therapy and education) to alleviate LEL symptoms with the goal of improving PF and the ability to perform ADLs.

Given that BMI is a risk factor associated with LEL and was associated with decreased PF and increased odds of needing help with ADLs, it was included as a confounder in multivariable models. We also considered that BMI may modify the association of LEL with PF and ADLs. However, no interaction association of BMI was observed. In terms of age, the findings of this study supported that age (ie, age 65-79 vs ≥80 years) modified the association between LEL and PF. The overall difference in PF between women who reported LEL vs those who did not was greater among women aged 80 years or older, which was consistent with the hypothesis that PF declines with age. Surprisingly, this pattern reversed among women who had colorectal cancer, such that the difference in PF was greater among women aged 65 to 79 years. This pattern underscores the disparate burden of LEL among survivors of colorectal cancer given that the negative association of LEL with PF exceeded age-associated PF decline. This suggests that clinical and behavioral interventions to prevent and improve LEL symptoms may need to be tailored by age group for survivors of colorectal cancer to maintain and improve PF.^37,51,52^ In addition, these findings suggest that future studies should focus on physiological mechanisms of LEL to understand why survivors of colorectal cancer experience different burdens of LEL in terms of age, PF, and ADLs.

This study has several notable strengths. First, this study had a large sample size of older female survivors of colorectal, endometrial, and ovarian cancer. This allowed the examination of LEL and its association with PF and ADLs by cancer type and allowed the advancing of the science about LEL among the understudied population of survivors of colorectal cancer. In addition, WHI LILAC used the recently developed and validated Lower Extremity Lymphedema Screening Questionnaire to assess clinically relevant LEL among women, which allows for clinical interpretations and comparisons across studies.^41^

### Limitations

This study has several limitations. The LILAC study relied on self-reported data, which may lead to recall and survivor bias. Because we relied solely on a self-reported LEL questionnaire, our results were generalizable to women who have symptomatic LEL. It is possible that women who had a diagnosis of mild LEL reported no LEL symptoms. Therefore, we may have underestimated the prevalence of LEL in the study population. Participants in the study had a higher mean age, with a longer time from cancer diagnosis (range, 1.42-20.23 years), and were more likely to be healthy compared with survivors of other cancers. This may have influenced study findings. Most women were White, insured, and college educated, which may also limit generalizability to the general cancer population. In addition, this study did not have access to detailed surgical information. It is possible that women who underwent aggressive surgical procedures were more likely to experience LEL, have a decline in PF, and need help with ADLs compared with those who underwent minimally invasive procedures. Moreover, other underlying diseases (eg, venous insufficiency) not due to cancer diagnosis or treatment may cause women to report edema rather than cancer-associated LEL. Because the timing of measuring LEL, PF, and ADLs varied within the sample, only associations could be determined, not causality of LEL, PF, and ADLs; in addition, given that this is an observational study, no causal conclusions can be made. Despite adjustment for potential confounders, there may be unmeasured confounders that biased observed associations.

## Conclusions

This study found that nearly one-third of older female survivors of colorectal, endometrial, and ovarian cancer reported LEL, which was associated with decreased PF and increased odds of needing help with ADLs. These findings suggest that clinicians may need to regularly assess the presence of LEL among survivors of colorectal, endometrial, and ovarian cancer. Future studies could further investigate physiological mechanisms associated with LEL, especially among patients with colorectal cancer, to investigate effective interventions associated with reduced burden of LEL on PF and ADLs for all survivors of cancer.

## Notes

Supplement. **eTable 1.** Demographic and Clinical Characteristics in Included and Excluded Participants From Life and Longevity After Cancer Study **eTable 2.** Interaction of Lower Extremity Lymphedema With Body Mass Index Category in Association With Outcomes
