# Supplementary material for: Association of Lower Extremity Lymphedema With Physical Functioning and Activities of Daily Living Among Older Survivors of Colorectal, Endometrial, and Ovarian Cancer
Source: JAMA Netw Open. 2022 Mar 9;5(3):e221671. doi: 10.1001/jamanetworkopen.2022.1671 (PMC8908072; doi:10.1001/jamanetworkopen.2022.1671)
Supplement: Supplement. — eTable 1. Demographic and Clinical Characteristics in Included and Excluded Participants From Life and Longevity After Cancer Study eTable 2. Interaction of Lower Extremity Lymphedema With Body Mass Index Category in Association With Outcomes [file jamanetwopen-e221671-s001.pdf]

Supplemental Online Content

Zhang X, McLaughlin EM, Krok-Schoen JL, et al. Association of lower extremity lymphedema with physical functioning and activities of daily living among older survivors of colorectal, endometrial, and ovarian cancer. *JAMA Netw Open*. 2022;5(3):e221671. doi:10.1001/jamanetworkopen.2022.1671

eTable 1. Demographic and Clinical Characteristics in Included and Excluded Participants From Life and Longevity After Cancer Study
eTable 2. Interaction of Lower Extremity Lymphedema With Body Mass Index Category in Association With Outcomes

This supplemental material has been provided by the authors to give readers additional information about their work.

© 2022 Zhang X et al. *JAMA Network Open.*

eTable 1. Demographic and Clinical Characteristics in Included and Excluded Participants From Life and Longevity After Cancer Study

Parameter	Level	LILAC (EC, CRC, OC[a]) N=1667	Included n=900	Excluded n=767	P-value
Age at form 370, years, mean ± SD		79.2 ± 6.1	78.5 ± 5.9	80.4 ± 6.2	<0.001
Race and Ethnicity[b], n (%)					
	White, non-Hispanic	1497 (89.8)	816 (90.7)	681 (88.8)	0.88
	Black or African American	70 (4.2)	36 (4.0)	34 (4.4)	
	Other[c]	100 (6.0)	48 (5.3)	52 (6.8)	
Marital Status, n (%)					
	Married or living as married	694 (45.9)	409 (47.6)	285 (41.9)	0.03
	Widowed	591 (38.4)	303 (35.2)	288 (42.3)	
	Divorced/separated	183 (11.9)	109 (12.7)	74 (10.9)	
	Never married	73 (4.7)	39 (4.5)	34 (5.0)	
Education, n (%)					
	College or Associate's Degree	1373 (83.3)	766 (85.4)	607 (80.7)	0.01
	High school/Less than high school	276 (16.7)	131 (14.6)	145 (19.3)	
Insurance, n (%)					
	Private	1070 (65.1)	626 (70.1)	444 (59.0)	<0.001
	Public	109 (6.6)	50 (5.6)	59 (7.9)	
	Public+Private	392 (23.8)	181 (20.3)	211 (20.3)	
	No Insurance	74 (4.5)	36 (4.0)	38 (5.1)	
BMI, kg/m^2, mean ± SD		26.9 ± 6.1	26.8 ± 6.0	26.9 ± 6.4	0.80
Cancer type, n (%)					
	Colorectal Cancer	818 (49.1)	421 (46.8)	397 (51.8)	0.08
	Endometrial Cancer	675 (40.5)	375 (41.7)	300 (39.1)	
	Ovarian Cancer	174 (10.4)	104 (11.6)	70 (9.1)	

[a]LILAC participants who had diagnosis of endometrial, colorectal, or ovarian cancer

[b]Race and Ethnicity were combined because of small sample sizes

[c]Other race including American Indian/Alaskan Native, Asian/Pacific Islander, Hispanic/Latino, Other not specified, and more than one race

© 2022 Zhang X et al. *JAMA Network Open.*

eTable 2. Interaction of Lower Extremity Lymphedema With Body Mass Index Category in Association With Outcomes

	Normal Weight (BMI<25 kg/m^2)		Overweight (BMI= 25-29.9 kg/m^2)		Obese (BMI≥30 kg/m^2)		P interaction[c]
	LS mean ± SE (95% CI)	P	LS mean ± SE (95% CI)	P	LS mean ± SE (95% CI)	P	
**PFS[a]**							
Overall	-15.2 ± 2.7 (-20.5, -9.9)	<0.0001	-17.0 ± 3.0 (-23.0, -11.1)	<0.0001	-18.6 ± 3.2 (-24.8, -12.4)	<0.0001	0.72
**By cancer site**							
Colorectal	-22.1 ± 4.2 (-30.4, -13.8)	<0.0001	-17.6 ± 4.3 (-26.1, -9.2)	<0.0001	-27.5 ± 5.1 (-37.5, -17.4)	<0.0001	0.33
Endometrial	-12.2 ± 4.4 (-20.8, -3.7)	0.0053	-16.7 ± 5.2 (-26.9, -6.5)	0.0014	-11.8 ± 4.5 (-20.7, -3.0)	0.0089	0.73
Ovarian	-3.8 ± 7.0 (-17.7, 10.2)	0.59	-22.0 ± 9.3 (-40.5, -3.6)	0.020	-21.9 ± 11.0 (-43.8, 0.0)	0.049	0.17
	Odds Ratio (95% CI)	P	Odds Ratio (95% CI)	P	Odds Ratio (95% CI)	P	P interaction[c]
**Need help with ADLs[b]**							
Overall	3.10 (1.62, 5.90)	0.0006	2.76 (1.35, 5.62)	0.005	2.22 (1.13, 4.39)	0.02	0.78
**By cancer site**							
Colorectal	4.25 (1.64, 11.00)	0.003	2.95 (1.13, 7.70)	0.03	6.46 (1.90, 21.92)	0.003	0.61
Endometrial	4.02 (1.36, 11.91)	0.012	1.49 (0.40, 5.58)	0.55	1.35 (0.53, 3.47)	0.53	0.30
Ovarian	0.35 (0.01, 10.49)	0.54	n/a[d]	n/a[d]	0.46 (0.01, 45.31)	0.74	0.98

[a]PFS: physical function score. Linear regression was used to estimate the mean difference in PFS between women who reported LEL and who did not report LEL. P-value for the 3-way interaction of LEL*BMI*cancer type is 0.38.
[b]ADLs: activities of daily living. Logistic regression was used to estimate the odds of needing help with ADLs between women who reported LEL and who did not report LEL. P-value for the 3-way interaction of LEL*BMI*cancer type is 0.89.
[c]Adjusted Wald test was used to test the interaction effect of LEL with BMI category.
[d]Small sample size of ovarian cancer limited the ability to calculate the effect estimates.
All models adjusted covariates including age, education, race and ethnicity, marital status, insurance type at enrollment, cancer stage at diagnosis, number of lymph nodes examined, time since diagnosis, self-reported chemotherapy, and self-reported radiation

© 2022 Zhang X et al. *JAMA Network Open.*
